# 3D Microlithography Using an Integrated System of 5-mm UV-LEDs with a Tilt-Rotational Sample Holder

**DOI:** 10.3390/mi11020157

**Published:** 2020-01-31

**Authors:** Sabera Fahmida Shiba, Hyeongmin Jeon, Jong-Soo Kim, Jong-Eun Kim, Jungkwun Kim

**Affiliations:** 1 Department of Electrical and Computer Engineering, Kansas State University, Manhattan, KS 66506, USA; sshiba@ksu.edu; 2 Samil Tech Co., LTD, Bucheon-si 14441, Korea; hmjeon89@naver.com (H.J.); davidkim605@gmail.com (J.-S.K.); samil@samiltech.com (J.-E.K.)

**Keywords:** Ultra Violet (UV) lithography, tilt rotational sample holder, SU8 microstructure, high aspect ratio, 3D microstructure

## Abstract

This paper demonstrates a 3D microlithography system where an array of 5 mm Ultra Violet-Light Emitting Diode (UV-LED) acts as a light source. The unit of the light source is a UV-LED, which comes with a length of about 8.9 mm and a diameter of 5 mm. The whole light source comprises 20 × 20 matrix of such 5 mm UV-LEDs giving a total number of 400 LEDs which makes it a very favorable source with a large area for having a batch production of the desired microstructures. This light source is able to give a level of precision in microfabrication which cannot be obtained using commercial 3D printers. The whole light source performs continuous rotational movement once it is turned on. This can also move up and down in a vertical direction. This multidirectional light source also comprises a multidirectional sample holder. The light source teaming up with the multidirectional sample holder highly facilitates the process of fabrication of a huge range of 3D structures. This article also describes the different levels of characterization of the system and demonstrates several fabricated 3D microstructures including high aspect ratio vertical micro towers, twisted turbine structures, triangles, inclined pillar ‘V’ structures, and hollow horn structures as well.

## 1. Introduction

**Figure 1:**
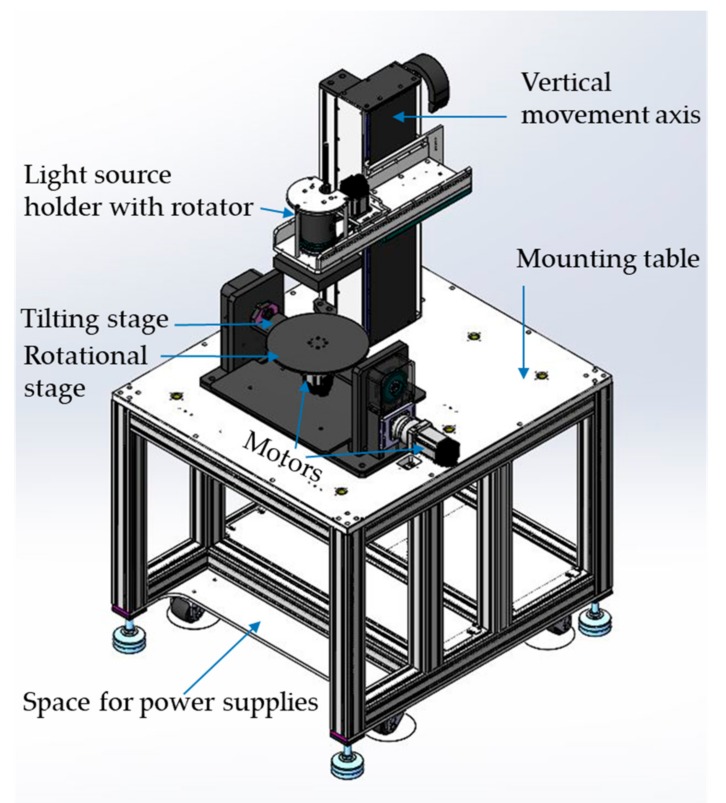
Demonstration of the UV-LED photolithography system.

The process of lithography has advanced manifolds with the course of time. Researchers have presented their findings and suggestions from time to time that have enriched the process of photolithography. A lithography technique has been presented where a single step of polyurethane methacrylate (PUMA) coating and two steps of UV exposure have been used to create a microchannel [1]. Soft X-ray has been exploited for fabricating microstructures where the sweet spot is considered for getting higher resolution structures [2]. A type of microfabrication process employs stereolithography and electroplating which is applicable to polymer and metal microstructures as well [3]. A proximity mode inclined lithography process has been discussed where a certain gap in between the mask and the substrate is maintained and it can be used for both front side exposure and backside exposure [4]. For some Bio-MEMS applications, electron beam lithography (EBL) has been used which is able to get sub-micron feature sizes [5]. A method of multi-beam interference for fabricating 3D polymeric microstructures has also been discussed [6]. UV Lithographie, Galvanoformung, Abformung (LIGA) technology is another method of fabrication for fabricating high-resolution microstructures where Lithographie, Galvanoformung and Abformung are the German acronyms for lithography, electroplating, and molding respectively [7]. The current fabrication procedure followed by different research groups is more or less similar in their applications [8] but the difference lies mainly in the light source used for exposure, the substrate holder used for determining the exposure direction, or the material used for making microstructures. Mercury vapor lamps have been in use as the UV exposure light source for decades. Commercially available mercury vapor lamps that contain mercury and xenon gases are even able to fabricate several millimeter range micro-pillars that can be used in an inductor and more other applications [9]. However, the mercury-vapor lamp emits light with several wavelengths that need to be filtered out. Also, this light source needs high maintenance and has a limited lifetime. Nowadays, LEDs are considered to be a proper replacement for these traditional gas discharge lamps. With the recent advancement of UV-LEDs and the availability of various wavelengths, researchers are moving towards an easier and more convenient UV-LED light source exposure system [10,11]. It has also been demonstrated how a specific wavelength of UV-LED can be a good replacement for traditional UV lamps that contains multiple-wavelength peaks [12] and can achieve success in maskless photolithography as well [13]. However, these UV-LED light sources were controlled manually and exposure is allowed only in the vertical direction. Also, they emphasized only small feature size patterns and none of them were reported to be able to fabricate millimeter range microstructures or 3D microstructures. Whereas the light source discussed here was able to fabricate both millimeter range microstructures and complex 3D microstructures. Choosing the material suitable for microfabrication is another important step of photolithography. Although positive photoresists like novolac and diazonaphthoquinone (DNQ) are still in use; however, SU8 negative photoresist is more popular for research and applications nowadays. A wide range of robust microstructures is fabricated using SU8 [14,15]. Straight pillars can be obtained from straight exposure under the UV light source. However, a lot of complex microstructures are needed for different applications. That depends on how the substrate is held under the light source. A lot of methods are there to fabricate them. One of the methods exploits the simple photolithography procedure, where the sample is exposed from backside with different angles and multiple exposures around the axes give high aspect ratio 3D structures as output [16]. Several exposure methods have been discussed where the stage or exposure angle should be controlled manually, which consumes a lot of time and lacks precision. The layering effect caused due to less precise control of the stage leaves the structure with rough surfaces and defects. The multidirectional movement of the substrate greatly facilitates complex structure fabrication and smooth surfacing [17]. When this multidirectional movement is automated and computer-controlled, there is greater ability to fabricate complex 3D microstructures. Employing two independent motors for controlling the rotational and tilting movement makes it possible to produce a wider range of complex microstructures [18]. The dynamic mode multidirectional UV light source was another step ahead in making advanced 3D structures where nanoscale range structures were successfully demonstrated [19]. Whereas a traditional UV light source was used for this system and the user had no control over the light source nature or intensity. Recent advancement includes the use of multidirectional UV-LEDs as light source that does not require high maintenance and can be switched on and off in certain areas of the substrate [20]. Substrate holders for these systems were separately set up with the light sources. There was no integrated system for both the light source control and substrate holder control. The exposure system discussed in this article is comprised of the user controllable light source with higher intensity and an integrated tilt rotational substrate holder, as well as dedicated software to run the programs. Figure 1 shows the drawing of the discussed microlithography system drawn in SolidWorks CAD software. There is a light source holder with a rotator where an array of LEDs is mounted. This holder along with LEDs is able to move up and down on the vertical movement axis with the help of a motor. On the mounting table, a substrate holder is placed with tilting and rotational stages which are operated by two motors respectively. The power supply accommodation is placed at the bottom of the mounting table. This infrastructure along with the computer control gives a commercial-grade setup which is able to give batch production of microstructures ranging from several microns to several millimeters. The light source discussed in this article is comprised of commercial 5 mm UV-LED and can be illuminated in certain areas and the part of the light source that needs to be illuminated is controlled by computer program.

## 2. System Analysis 

### 2.1. Light Source 

**Figure 2:**
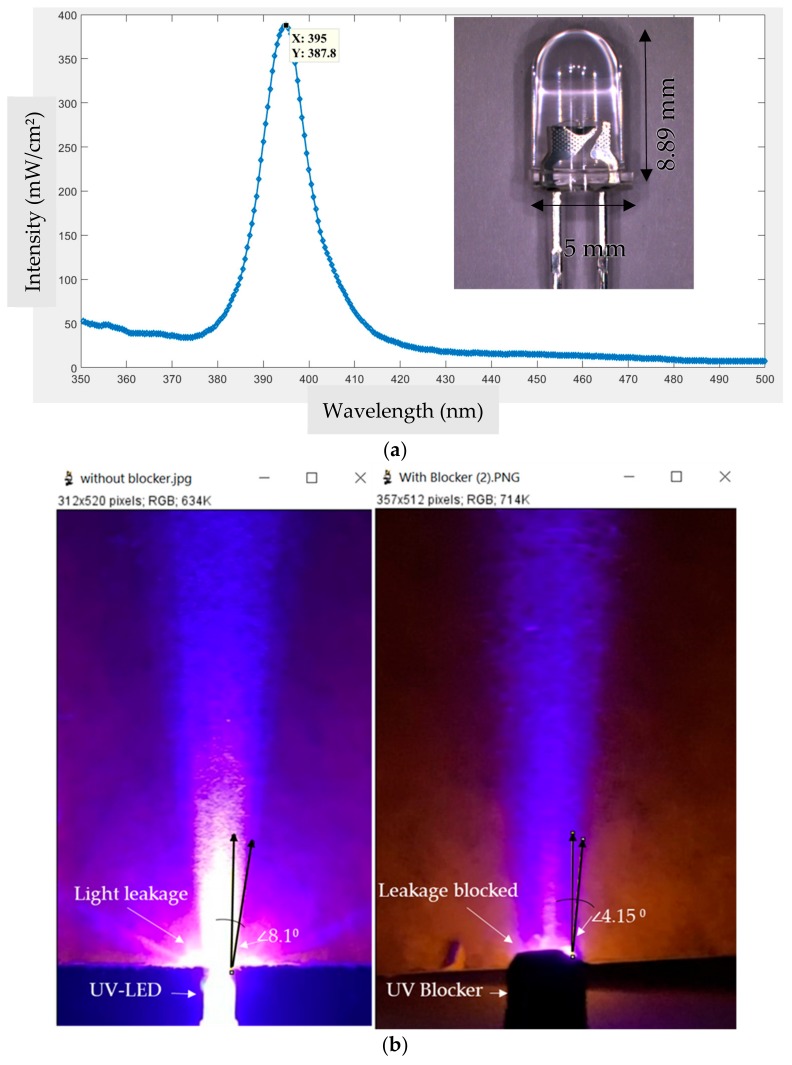
Light source analysis: (**a**) UV-LED wavelength Spectrum (inset: The 5 mm UV-LED with parameters); (**b**) Comparison of the UV light leakage and collimation with and without the UV blocker.

A variety of LEDs are available commercially. They are usually differentiated by wavelength, color, and other parameters. The unit of the exposure system that has been discussed here is an LED with a 5 mm diameter. This UV-LED emits light with a wavelength of 395 nanometers. The spectrum of this LED was analyzed by using an optical spectrum analyzer (BLUE-Wave, StellarNet Inc., Tampa, FL, USA). Figure 2a shows the graph plotted in MATLAB obtained from the spectrum analysis, where the *x*-axis represents the wavelength in the nanometer, and the *y*-axis represents the intensity measured in mW/cm². Half of the intensity bandwidth is covered in between the range of 390 nm to 410 nm. As the peak indicates a unique value at 395 nm with an intensity of 387.8 mW/cm², there is no necessity to filter out any other spectra. The inset of Figure 2a shows the 5 mm UV-LED which is the unit of the light source of this system.

Figure 2b shows how light leakage is minimized by using a UV blocker. As the UV-LEDs in this system are placed very close to each other, the adjacent light beams interfere with each other and hamper the process of microlithography. To block this interference and direct the light beams in nearly collimating direction, an opaque UV blocker has been used surrounding each LED as shown in Figure 2b. The central beam shows an angular deviation of 8.1 degrees from the normal position whereas, after using the blocker, the angular deviation reduces to 4.15 degrees which is acceptable for microlithography.

Each LED has a circular illuminating region, where the 400 LEDs cover an illuminating area of 4 square inches on the PCB. As an individual LED covers a circular region, they leave a certain gap with the adjacent circular LEDs. If the LEDs were arranged in regular matrix format the adjacent LEDs would leave an almost rectangular shaped gap. This gap is supposed to leave a large dark area in between the adjacent illuminated areas, which is not favorable for SU8 microfabrication. In order to minimize this gap in between the adjacent column and row, the LEDs have been arranged in the zigzag format so that the large rectangular gap is covered by the LED from the following row. The light source is made to rotate continuously so that the gaps are covered in the path of rotation and a more uniform illuminated path is obtained. LEDs are mounted on the PCB. The UV blocker was made slightly higher than the LEDs so the diffractions were eliminated and the light rays were directed towards the target.

### 2.2. Software

In order to make 3D structures of desired shapes, the rotation and the tilting should be controlled according to the users’ preferences. The C+ program has been employed to control both the rotational angle and the tilting angle. Certain movement of the stepper motor is equivalent to a certain angle of rotation or movement. The software is used to predefine the movement angles as well as the speed of movement. One advantageous prospect for the structures fabricated is that the models are not designed in 3D CAD modeling and uploaded to the software. Rather, tilting and rotational motion directions are visualized or predicted as per the user’s desired 3D structure design. Then the exposure angle and the speed for the motions are predefined using the C+ program which would most likely suit the user’s applications. Therefore, no additional skill is needed in 3D CAD modeling or algorithm languages.

### 2.3. System Setup

The base of the light source contains a heat sink and cooling fans so that the heat created by the light source is under control. The light source along with the base was suspended at the free end of a metal cylindrical chuck.

**Figure 3:**
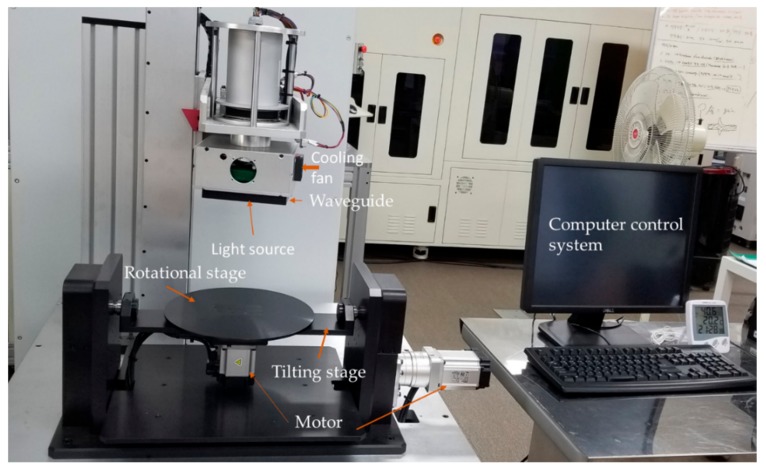
Overall system setup for the exposure system along with the computer control system.

The electrical connections are housed securely inside a long cylindrical base. Figure 3 shows an overall picture of the machine setup. The substrate holder used along with the light source is able to give both rotational and tilting movement. Two stepper motors are employed to control tilting and rotational movements, respectively. The tilting and rotational movement enable the substrate to be placed at a wide range of angles with respect to the light source. The substrate holder is aligned well with the light source above. The power supplies were housed below the substrate holder table. The computer was set beside the machine setup for convenient control for the user. The computer control, the light source, and the tilting rotational substrate holder all work to synchronize with the user’s preference.

## 3. Fabrication Method

**Figure 4:**
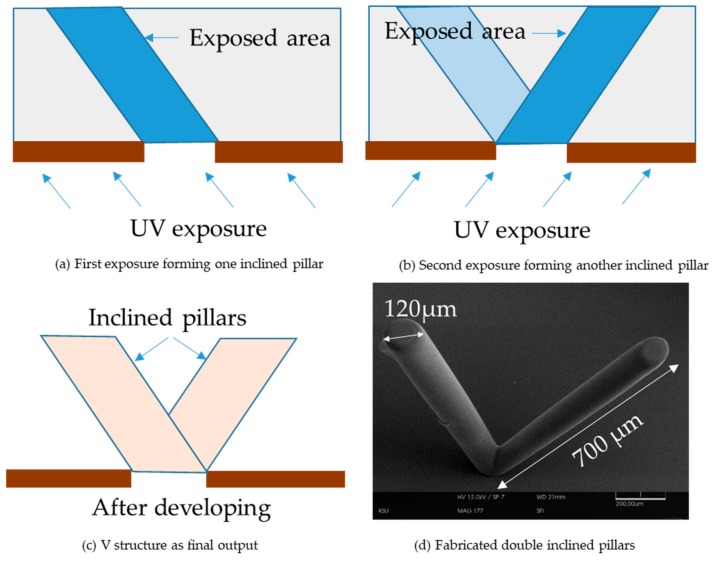
Fabrication procedure: (**a**) First inclined pillar formation; (**b**) Second pillar formation; (**c**) ‘V’ like double inclined pillars as output after developing; (**d**) SEM image of a ‘V’ structure fabricated using the UV microlithography system.

In order to fabricate microstructures, a substrate needs to be photo-patterned first. Usually, the desired photo-patterns are designed in AutoCAD (2017, Autodesk, San Rafael, CA, USA) and projected on a 1 × 1 inch chromium coated glass (Telic Company, Valencia, CA, USA). The samples demonstrated in this paper were the Chromium coated glasses which were spin-coated later with S-1805 (a positive photoresist) with a speed of 3000 rpm followed by soft baking for 1 min on a hot plate at a temperature of 95 degrees. Then the coated substrate was photo-patterned using the projection lithography. Then was developed using MF 319 developing solution (Dow Chemical Company, Marlborough, MA, USA). Then, after checking the patterns using the microscope, the sample was etched using Chromium etchant so that the Chromium coated part of the exposed part of the glass goes away and leaves the desired patterns to be transparent. Then organic cleaning with acetone, methanol, and isopropanol was done so that the S-1805 coating goes away. After that, the weight of poured SU8 (negative photoresist) was measured before soft baking, and the corresponding height of the SU8 was noted after soft baking. A linear relationship between the weight of SU8 and the height of SU8 was obtained and was followed for controlling the heights of SU8. For these fabrications, SU8 2025 was used which contains about 68.55% solids. As this SU8 has a higher viscosity (4500 Centi-Stokes) and was spread uniformly over a larger area, the surface tension does not affect much in height variation. Here, the height of the baked SU8 was considered rather than the height in liquid or semi-liquid state. For demonstration purposes, one ‘V’ structure has been shown here with the fabrication procedure details. The sample was kept tilted at a +60 degree at a distance of 20 mm for 1 h that gives one inclined pillar, which has been demonstrated in Figure 4a. After that, the sample was tilted to −60 degrees and was exposed for one more hour. This gives the second inclined pillar, which has been shown in Figure 4b,c shows the double inclined pillars that are supposed to be obtained after developing. Figure 4d shows the SEM image of the fabricated double inclined pillar structure fabricated using the explained procedure. No rotational movement was involved in this process. As for tilting the stage, the slit or the photo pattern size becomes smaller as seen from the light source. Less light can pass through the narrower patterns and as a result, the cured depth is decreased. To achieve equivalent vertical cured depth, the exposure dose needs to be increased.

Vertical pillars take almost half of this exposure time to fabricate. After exposing, the sample was baked at 95 degrees on a hot plate for 50 min, which is post-exposure baking and it helps the cross-linking to be completed. Then for getting rid of the unexposed SU8, the sample was developed in a fresh developer for 23 min. This is a simple example of the fabrication procedures followed for the 3D structures discussed. However, the fabrication of the 3D structures is totally dependent on the UV light exposure. Therefore, this method is able to fabricate structures that start growing from the base photo patterns. It is not able to fabricate structures like hanging or bridging structures where the light cannot pass through from the pattern. For example, it can fabricate structures like ‘V’ as the light can pass continuously from the bottom to top with a definite slope, but it cannot fabricate structures like ‘Z,’ ‘A’, or ‘T’ as the hanging or bridging structures are not able to collect exposure from the photo patterns.

## 4. Characterization

### 4.1. Versatility

For lithographic microfabrication, different exposure doses are required for different applications. As the exposure dose is directly dependent upon the light intensity, the light intensity measurement is of paramount importance. The exposure system explained above was characterized by intensity measurements at different DC current levels and at different distances.

**Figure 5:**
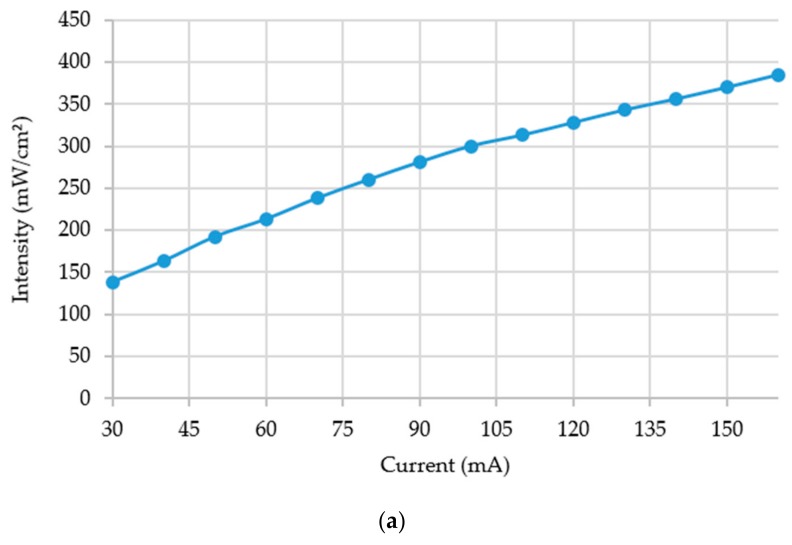

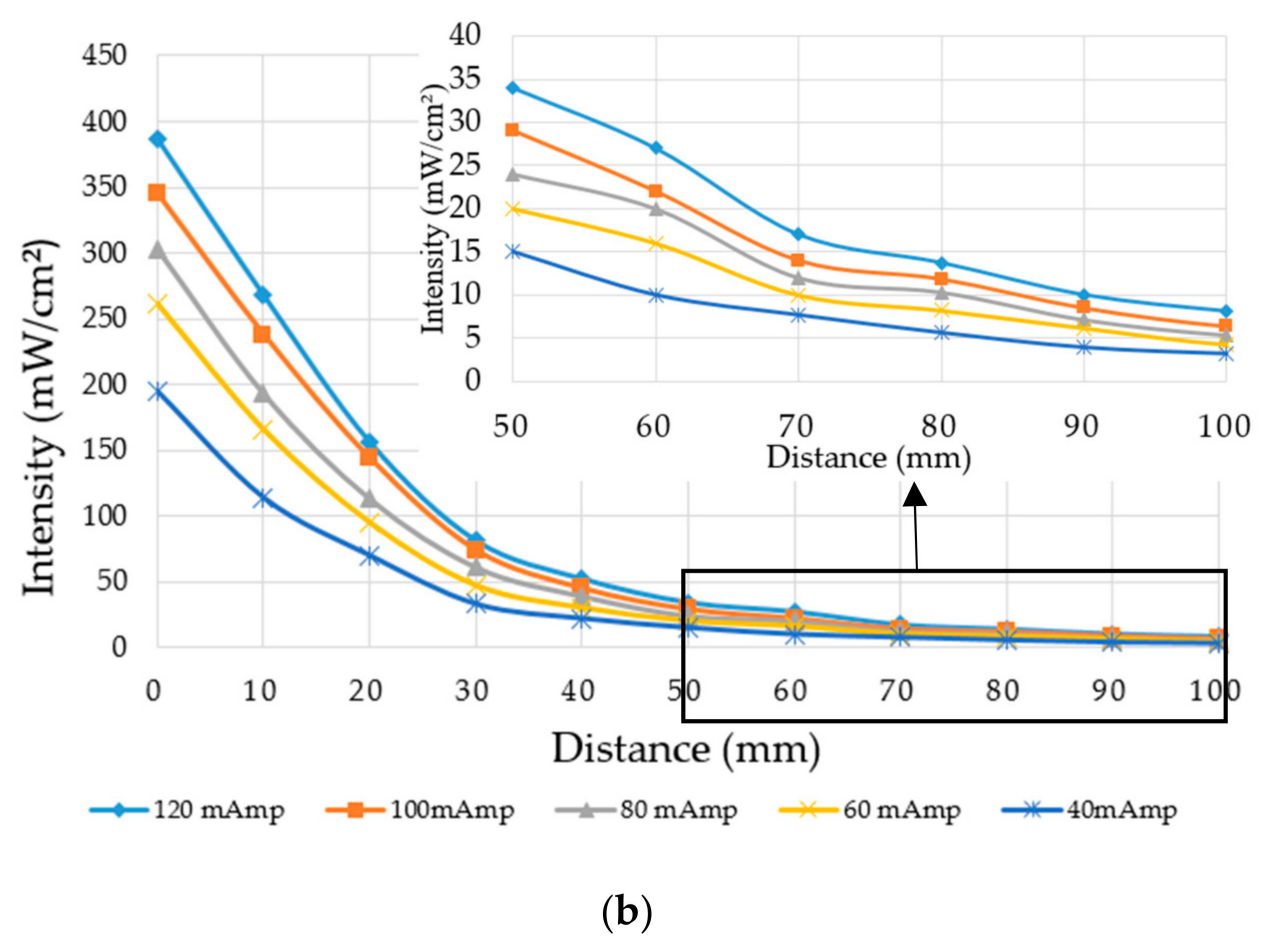
Intensity variation: (**a**) Intensity variation with the change of DC current applied on a single LED. (**b**) Intensity variation with the change of distance at different levels of DC current (inset: intensity variation within the range of 50 mm to 110 mm distance from the light source).

Figure 5a shows a graph demonstrating a linear relationship between intensity and DC current. The *x*-axis represents the DC current applied in milliampere whereas the *y*-axis gives the respective intensity in milliwatts per square centimeter. Figure 5b shows an exponential relationship between intensity and linear distance between the LED and the intensity meter. As the distance gets longer the intensity becomes less and less but strong enough to accomplish microlithography exposure. The inset of the figure shows the variation of intensity for further distances between 50 mm and 110 mm. A very significant intensity is observed even at such far away distances from the light source.

### 4.2. Reliability

**Figure 6:**
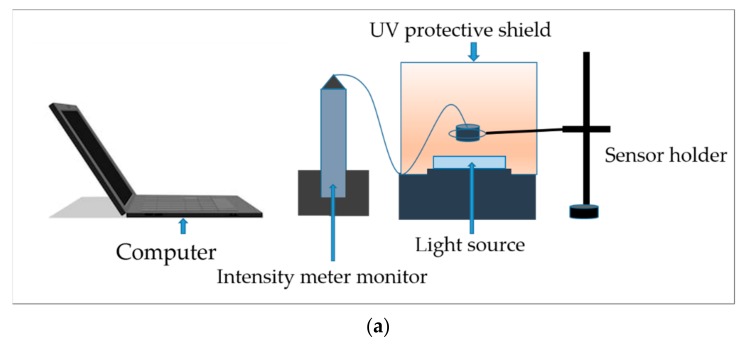

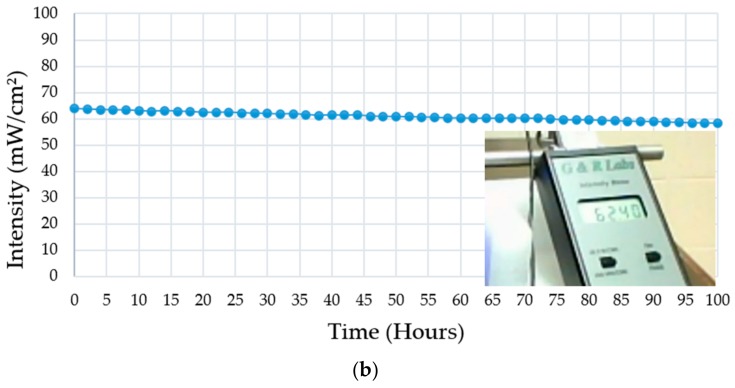
Light source reliability test: (**a**) Experimental setup with monitoring system; (**b**) Intensity data with time variation.

Long-time exposure is needed in order to get tall pillars and complex 3D structures. As a result, the light is required to keep turned on for several hours. So, the light should maintain a stable intensity for a long time. Figure 6a shows the setup for measuring light intensity over 100 h. In this job, a software named i-Spyconnect (2018) was employed to monitor and record the intensity for this whole time. The sensor of the intensity meter was placed above the light source.

The intensity meter was placed facing the computer monitor so the values of the intensity could be recorded. Figure 6b shows data plotted on a graph where *x*-axis plots time and the *y*-axis represents the intensity measured.

## 5. Results

**Figure 7:**
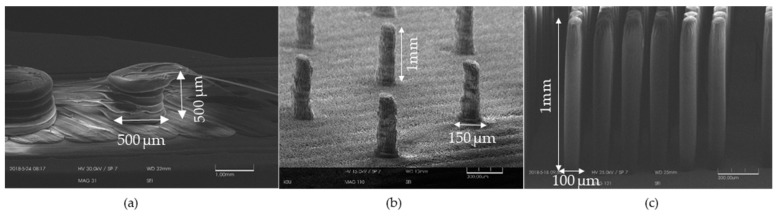
Micro pillars fabrication comparison using different systems (Diameter 100 µm and height 1000 µm): (**a**) Micro pillars printed using fused deposition modeling (FDM) 3D printer, giving a diameter of 500 µm and a height of 500 µm Poly-Lactic Acid (PLA)pillar array; (**b**) Micro pillars printed using Formlabs 2 stereolithography (SLA) printer giving a diameter of 150 µm and height of 1000 µm; (**c**) Fabricated pillars using the discussed light source giving around 100 microns diameter and 1000 microns height and smooth surface.

A comparison has been made between the pillars fabricated using the discussed light source and the structures printed by different 3D printers that are available commercially. Both fused deposition modeling (FDM) printer (XYZ printing, Lake Forest, CA, USA) and stereo-lithography (SLA) printer (Formlabs, Sommerville, MA, USA were employed to get micro-pillars with a high aspect ratio. Figure 7a shows the FDM printed pillars that fail to print after only 500 microns of printing. Figure 7b shows the SLA printed structures that exhibit layering effects on the structures. Side by side, Figure 7c shows the smooth surfaces obtained from the light source discussed above.

**Figure 8:**
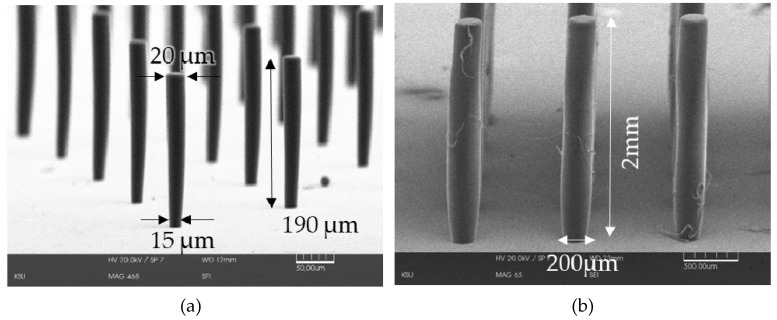
Versatility of the lithographic system: (**a**) 1:12 micro-pillars with 15 µm photo-pattern; (**b**) 1:10 micro-pillars with 200 µm photo patterns.

The system does not only give smooth surfaces but also is able to fabricate a wide range of microstructure. It is able to fabricate micron-level precision as well as millimeter range of micro-pillars. And these high aspect ratio pillars have potential use as monopole RF antennas. Figure 8 shows an example of the versatility of the discussed lithographic system as Figure 8a shows 1:12 aspect ratio micro-pillars standing on a base of only 15 microns and Figure 8b shows 1:10 aspect ratio 2-millimeter high vertical pillars. The top parts of the 15-micron pillars are 5 microns wider than the bottom as the sample was slightly over-exposed than required.

**Figure 9:**
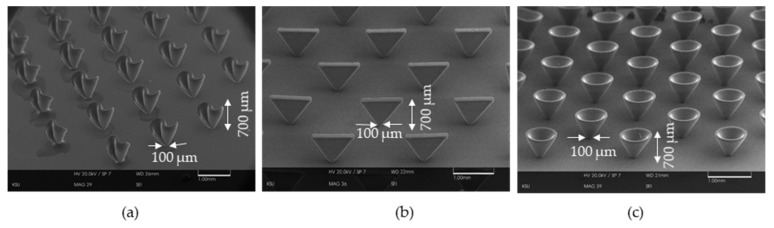
3D microstructure array: (**a**) Array of twisted micro turbine structure array; (**b**) Array of vertical triangle structures; (**c**) Array of hollow horn structures.

Some microstructures fabricated using the proposed light source has been demonstrated here. The structures fabricated here have real values for length, width, and heights. The structures are not just projected versions of flat 2D images or 2.5D images but they are clearly 3D structures suitable for scientific applications like micro antenna fabrication, biosensor applications. Figure 9a shows a twisted microstructure with a base diameter of 100 microns and it gives a height of around 700 microns. The pattern size varied between 100 microns to 139 microns all over the substrate. The sample was exposed keeping it tilted at 70 degrees and the rotational disk was rotated back and forth between +150 degrees and −150 degrees at a speed of 2500 degrees per second. The exposure time was 3 h. This structure can be applied in microturbine systems.

For making straight pillar-like structures just straight exposure works keeping the substrate straight under the light source. However, for achieving complex microstructures like horns, inclined pillars, or twisted structures, rotational or tilting substrate holder needs to be used. Figure 9b shows an array of triangle 3D structure with a bottom thickness of 100 microns and around 700 microns in height. To fabricate this structure, the SU8 coated substrate was kept tilting back and forth between +60 degrees to −60 degrees. No rotation was involved in this exposure. The highest level of exposure was employed for 3 h at a vertical distance of 20 mm from the light source. After that, post-exposure baking was done for 50 min and developing in fresh developer solution for 25 min. This simple structure can be used in micro antenna systems which are commonly addressed as bow-tie antenna. The horn structures shown in Figure 9c were fabricated on a mask of 100-micron patterns and an applied SU8 thickness of 700 microns. The sample was kept inclined at an angle of 60 degrees at a distance of 2 cm. Then, a continuous rotation of 100 rpm was applied. The exposure intensity was 20 mW/cm². The exposure time was 3 h. Post-exposure baking was done for 50 min at a temperature of 95 degrees. The developing time was considered to be 25 min. The very smooth finishing of the structures and the variety of 3D microstructures demonstrated here shows the ability of the light source as a well-structured and well-designed microlithography system. The horn structures shown in the above figure hold the potential to be used as micro horn antennas.

## 6. Conclusions

Computer-controlled UV-LED lithography has been successfully implemented for 3D microfabrication where an array of UV-LED with a tilt-rotational sample holder can create a variety of 3D light traces by varying the light intensity as well as the exposure angles in a computer programmed way. The UV-LED light source explained here is capable of operating at a high-intensity level and a variable range of intensities. This light source is also able to give a batch production of microstructures as various examples have already been shown. The automated movement of the tilt rotational structure has given this light source an ability to fabricate thousands of different 3D microstructures which have great potential in the microelectronics field, as well as biosensor applications. The smooth finishing of the microstructures are better than any commercial 3D printers and this feature can be unique for being commercial UV photolithographic light source. The light source is reliable for using continuously for a whole lot of time with constant high intensity. The simultaneous vertical and rotational movement of the light source centering the substrate holder is an exceptional feature, as well as the precisely controlled movement of the substrate holder. The system is reported to be able to fabricate as small as 3 microns feature size resolution and micro towers as tall as 2 mm with an aspect ratio of 1:10. So far, the gap between adjacent patterns has been up to 30 microns, which can be changed to other values. The system discussed has a lot of improvements regarding precision, batch production, high surface quality, and greater variety of production; whereas its application is limited to UV exposable fabrications only. This system lacks the capability of fabricating macro structures or unsupported structures. As the intensity is high and variable, it consumes less time and is flexible compared to the traditional systems. As UV-LEDs are known to be simple and low-cost maintenance, the proposed 3D UV-LED lithography system will be highly competitive to the conventional systems with the advanced automated system as previously described. The integrated system of the multidirectional UV-LED light source along with the multidirectional substrate holder has been successfully developed as commercial-grade which is capable of both traditional lithographic fabrications and advanced 3D microfabrication, fulfilling the growing demand of an RF and bio-applications such as 3D micro-machined antennas or tissue-mimicking scaffold.
